# Enrichment and Genome Sequence of the Group I.1a Ammonia-Oxidizing Archaeon “*Ca*. Nitrosotenuis uzonensis” Representing a Clade Globally Distributed in Thermal Habitats

**DOI:** 10.1371/journal.pone.0080835

**Published:** 2013-11-20

**Authors:** Elena V. Lebedeva, Roland Hatzenpichler, Eric Pelletier, Nathalie Schuster, Sandra Hauzmayer, Aleksandr Bulaev, Nadezhda V. Grigor’eva, Alexander Galushko, Markus Schmid, Marton Palatinszky, Denis Le Paslier, Holger Daims, Michael Wagner

**Affiliations:** 1 Winogradsky Institute of Microbiology, Russian Academy of Sciences, Moscow, Russia; 2 Department of Microbiology and Ecosystem Science, University of Vienna, Vienna, Austria; 3 Commissariat à l’Energie Atomique, Genoscope, Evry, France; 4 Centre National de la Recherche Scientifique, Evry, France; 5 Université d’Evry-Val-d’Essonne, Evry, France; University of Zurich, Switzerland

## Abstract

The discovery of ammonia-oxidizing archaea (AOA) of the phylum *Thaumarchaeota* and the high abundance of archaeal ammonia monooxygenase subunit A encoding gene sequences in many environments have extended our perception of nitrifying microbial communities. Moreover, AOA are the only aerobic ammonia oxidizers known to be active in geothermal environments. Molecular data indicate that in many globally distributed terrestrial high-temperature habits a thaumarchaeotal lineage within the *Nitrosopumilus* cluster (also called “marine” group I.1a) thrives, but these microbes have neither been isolated from these systems nor functionally characterized *in situ* yet. In this study, we report on the enrichment and genomic characterization of a representative of this lineage from a thermal spring in Kamchatka. This thaumarchaeote, provisionally classified as “*Candidatus* Nitrosotenuis uzonensis”, is a moderately thermophilic, non-halophilic, chemolithoautotrophic ammonia oxidizer. The nearly complete genome sequence (assembled into a single scaffold) of this AOA confirmed the presence of the typical thaumarchaeotal pathways for ammonia oxidation and carbon fixation, and indicated its ability to produce coenzyme F_420_ and to chemotactically react to its environment. Interestingly, like members of the genus *Nitrosoarchaeum*, “*Candidatus* N. uzonensis” also possesses a putative artubulin-encoding gene. Genome comparisons to related AOA with available genome sequences confirmed that the newly cultured AOA has an average nucleotide identity far below the species threshold and revealed a substantial degree of genomic plasticity with unique genomic regions in “*Ca*. N. uzonensis”, which potentially include genetic determinants of ecological niche differentiation.

## Introduction

The discovery of ammonia-oxidizing archaea revealed a new microbial group involved in nitrogen cycling [1-3]. All AOA recognized today are members of the phylum *Thaumarchaeota* [4,5], whose representatives in culture share ammonia oxidation as their only demonstrated pathway for energy conservation. However, at least some members of this phylum can obtain in the environment energy for growth from yet unidentified substrates other than ammonia [6]. While until recently research on the first step of the nitrification process in the environment mainly focused on ammonia-oxidizing bacteria (AOB), which thrive in most terrestrial and aquatic systems [7,8], it has been shown during the last decade that thaumarchaeotes outnumber AOB in many ecosystems, including some soils [9–12], oceanic waters [13–15] and sediments [16,17].

In contrast to AOB, AOA also play a crucial role for nitrification in high temperature habitats [18-20]. In hot springs where ammonia is an abundant electron donor for chemolithotrophy high overall potential energy yields can be obtained from ammonia oxidation [21]. Enrichment cultures of thermophilic (at 74°C) [18] and moderately thermophilic (at 46°C) [22] AOA have become available, and the widespread presence of archaeal ammonia monooxygenase subunit A (*amoA*)-like genes in high temperature habitats up to 97°C has been demonstrated. So far, such *amoA*-like gene sequences have been found in subsurface thermal springs [23,24], many terrestrial hot springs [19,20,25-27] and deep-sea hydrothermal vents [28,29]. Measurements of *in situ* nitrification [20,27] and of *amoA* gene transcription [19,30] in several hot springs further indicate an important role of thermophilic AOA in these systems. The presence of crenarchaeol (more accurately named thaumarchaeol [5]), which is a signature lipid component of both mesophilic [31,32] and thermophilic AOA [18,33], in terrestrial hot springs lends additional support to the importance of AOA in thermal habitats [34-37].

Despite the global distribution and ecological significance of AOA, knowledge of the physiology and genomic make-up of these organisms are still relatively sparse. This knowledge gap can partly be attributed to the difficulties of cultivating the slow-growing AOA under laboratory conditions. Thus, only few enrichment or pure cultures of AOA are currently available [18,22,38-44].

In this study, we inoculated mineral medium with samples from a hot spring located in the Uzon caldera on the Kamchatka peninsula (Russia). Stable ammonia-oxidizing enrichment cultures were established and grown at 46°C. We present evidence that in one of these cultures a novel, moderately thermophilic AOA strain is solely responsible for the observed oxidation of ammonia. We demonstrate that this thaumarchaeote, which we designate as “*Candidatus* Nitrosotenuis uzonensis”, represents a lineage of AOA that is globally distributed in terrestrial high temperature environments, but was not brought into culture previously. Furthermore, a near-complete genome sequence of “*Candidatus* Nitrosotenuis uzonensis” was obtained and we demonstrate the presence of (i) genes typically found in chemolithoautotrophic thaumarchaeotes, (ii) provide evidence for flagellation and chemotaxis, and (iii) confirm the status of “*Candidatus* Nitrosotenuis uzonensis” as a new species and member of a new genus by average nucleotide identity analyses.

## Results and Discussion

### An ammonia-oxidizing enrichment culture from a hot spring in the Uzon caldera

**Figure 1:**
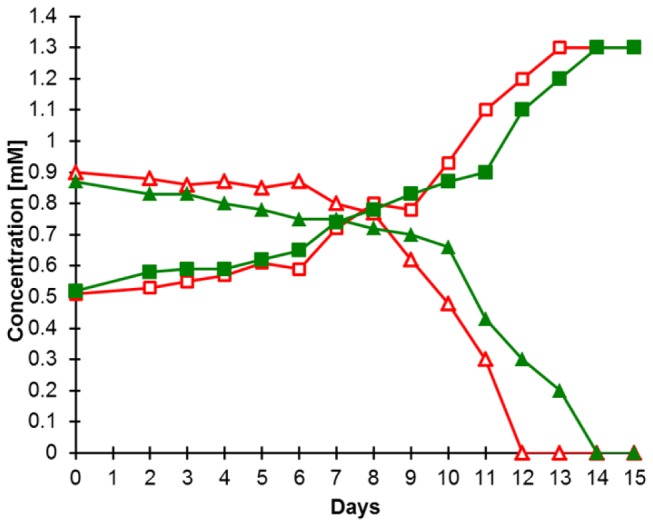
Near-stoichiometric oxidation of ammonia and production of nitrite by culture N4 over 15 days. Two replicate culture flasks (indicated by red and green symbols) were inoculated with 10 vol% of the parent culture, leading to an initial nitrite concentration of ~0.5 mM. Nitrate was not detectable during the whole experiment. Axis scaling is identical for the ammonia (triangles) and nitrite (squares) concentrations.

An ammonia-oxidizing enrichment culture seeded with material from a geothermal spring located in the Uzon caldera on the Kamchatka peninsula (Russia) was established and maintained in the laboratory for seven years. During the initial enrichment phase, different incubation temperatures were tested and the enrichment was observed to oxidize ammonia within a range of 28-52°C. For all further incubations 46°C was chosen, because it resulted in the highest ammonia removal rates. In later stages of the enrichment nitrite accumulated (Figure 1) and no nitrate was produced, indicating the absence of active nitrite-oxidizing bacteria (NOB). A parallel enrichment of NOB from the same primary enrichment recently resulted in the isolation of a novel thermophilic member of the genus *Nitrospira* [45].

A highly enriched AOA culture, in the following referred to as “culture N4”, was obtained by consecutive application of vancomycin (100 mg L^-1^) and streptomycin (50 mg L^-1^) followed by inoculation into fresh medium (10 vol% of the culture). This procedure was repeated three times and subsequently the culture was further purified by repeated subculturing from diluted suspensions. At the time of manuscript submission, only one morphotype was visible in the enrichment culture by phase microscopy, but PCR screening with three different primer sets (Table S10 in File S1) targeting bacterial 16S rRNA genes revealed a cryptic bacterial contamination as these primer pairs still yielded an amplification product of the expected size. The very low number of bacterial contaminants was confirmed by fluorescence in situ hybridization (FISH) as no signals were observed after hybridization with the EUB338 probe set targeting almost all members of the bacteria (data not shown). Notably, ammonia oxidation of culture N4 was inhibited by addition of bacitracin (100 mg L^-1^) and rifampicin (20 mg L^-1^). While most archaea are resistant to rifampicin, many rifampicin-sensitive halobacterial and methanogenic species have been reported [46,47]. For some of these archaea a detergent-like effect of rifampicin has been suggested [27]. The addition of low concentrations (20 mg L^-1^) of organic substrates, namely formate, acetate, succinate, and pyruvate (shown to have a growth-stimulating effect on *N. viennensis* EN76) [44] did not result in a greater rate of ammonia oxidation as compared to purely chemolithoautotrophic conditions (data not shown). Addition of HEPES-buffer (20 mM) to the growth medium inhibited ammonia oxidation. Culture N4 oxidized ammonia in the range of 0.05-1.0 g NaCl l^-1^ salinity with an optimum at 0.5 g salinity (data not shown). In contrast to the primary enrichment, culture N4 ceased to oxidize ammonia in the CaCl_2_-based medium as soon as the pH dropped below 6 (for comparison the pH at the sampling site was 6.5). Re-adjusting the pH did not restore ammonia-oxidizing activity. Consequently, culture N4 was till then maintained exclusively in CaCO_3_-based medium.

### Absence of ammonia-oxidizing bacteria

Several molecular methods were applied to screen culture N4 for the presence of known AOB. Bacterial 16S rRNA genes were PCR-amplified with the broad-coverage primers 616F and 1492R (Table S1 in File S1) from the enrichment, cloned, sequenced, and subjected to phylogenetic analysis. None of the 30 analyzed bacterial 16S rRNA gene clones was affiliated to known AOB (data not shown). Consistent with these results, all attempts to PCR-amplify bacterial *amoA* genes from this culture were unsuccessful even when up to 40 PCR cycles were used. Similarly, no AOB were detected by fluorescence in situ hybridization (FISH) using established rRNA-targeted probes that are specific for betaproteobacterial AOB (Table S2 in File S1). These results are consistent with the absence of genomic sequences from AOB in the obtained metagenomic dataset (see below).

### Molecular detection of a novel ammonia-oxidizing thaumarchaeote

**Figure 2:**
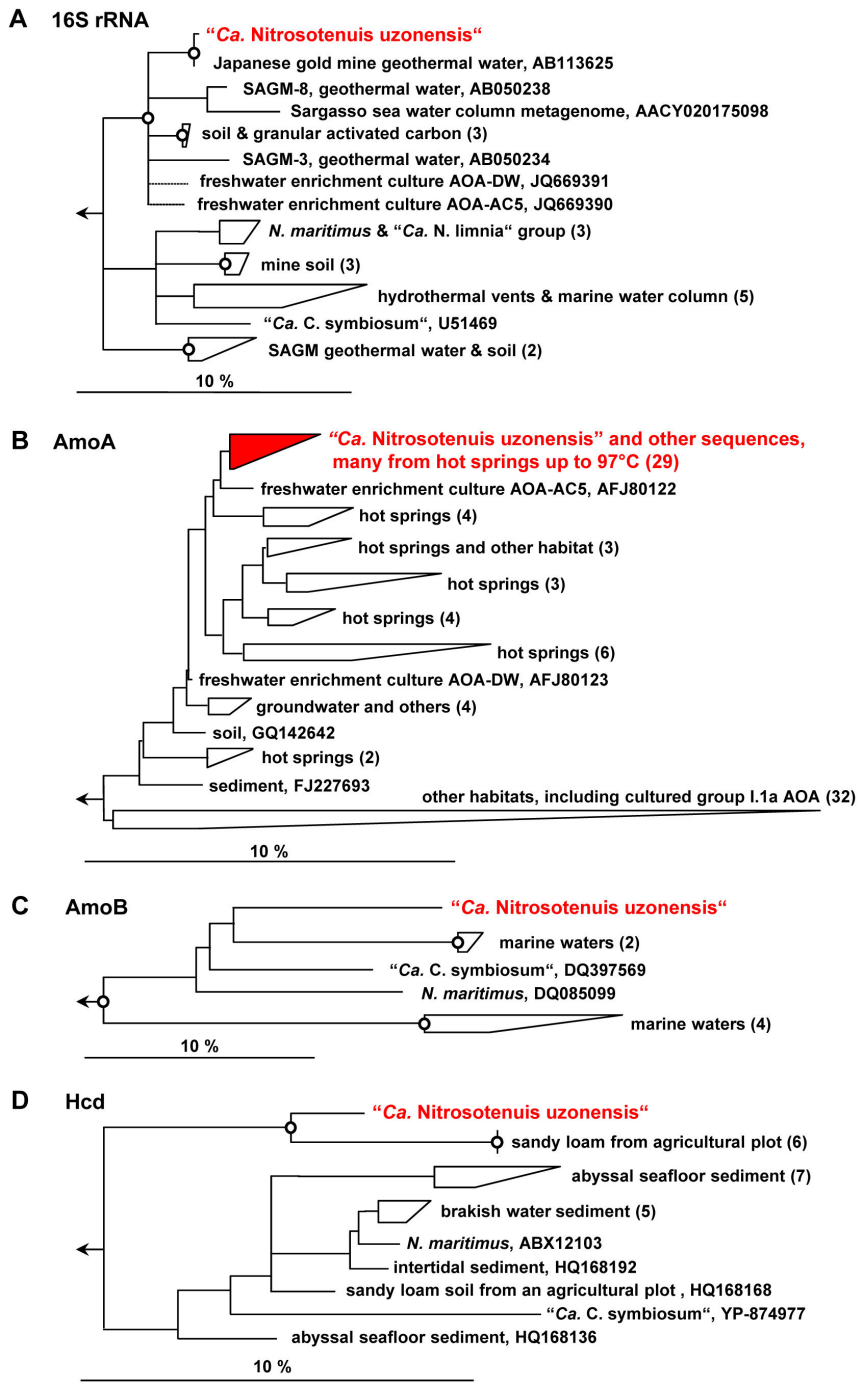
Phylogenetic trees inferred from the 16S rRNA (A), AmoA (B), AmoB (C) and Hcd (D) sequences of “*Ca*. Nitrosotenuis uzonensis“ and related archaea. All trees show only *Nitrosopumilus* cluster (also called group I.1a) sequences, while the respective sequences from the *Nitrososphaera* cluster (also called group I.1b), *Nitrosotalea* cluster (group I.1a-affiliated) and *Nitrosocaldus* cluster (ThAOA group) are contained within the outgroups. Circles on tree nodes indicate parsimony bootstrap support ≥90%. Numbers in parentheses indicate the number of sequences within a group. Dotted lines were used for short sequences that were added after construction of the overall tree. Scale bars show 10% estimated sequence divergence. SAGM, South African Gold Mine. For an extended AmoA tree please refer to Figure S1.

During the early stages of culture N4 an archaeal 16S rRNA gene library was established using primers 21F and 1492R (Table S1 in File S1). Eight clones were randomly selected and sequenced. At a later stage of the culture, a second archaeal 16S rRNA gene library was established. Screening of 31 clones from this library by restriction fragment length polymorphism (RFLP) analysis yielded three band patterns. Ten nearly full-length 16S rRNA sequences representing these patterns were further analysed. All sequences obtained from these two libraries were highly similar to each other (>99.0%), fell within the *Nitrosopumilus* cluster [48] (also called group I.1a) of *Thaumarchaeota*, and were related to the 16S rRNA of the recently enriched strains “AOA-AC5” and “AOA-DW” obtained from freshwater sediment (~97% identity over the only partially [~800 nt] available 16S rRNA gene of these freshwater AOA) [39]. The next closest relative (for which a full sequence is available) was *Nitrosopumilus maritimus* SCM1 (92.8% identity; Figure 2A).

Clone libraries were also established for archaeal *amoA* and *amoB* (ammonia monooxygenase subunits A and B) genes, and 39 and 12 clones, respectively, were randomly picked and sequenced. All obtained *amoA* and *amoB* gene sequences were highly similar (≥99.1% nucleotide identity for each gene) and clearly differed from those of other identified AOA (Figure 2B, C), with enrichment “AOA-AC5” as the closest cultured relative (92% nucleotide identity of the *amoA* gene; *amoB* is not available for this strain). Intriguingly, the *amoA* sequence of the novel thaumarchaeote is closely related to sequences that have been obtained from various geographically distant high temperature terrestrial environments (Figure 2B and Figure S1), such as Austrian subsurface radioactive thermal springs [49], geothermal mine waters in the United States [23], and numerous hot springs in Asia, North America, and Iceland, which exhibit a wide range of physicochemical parameters (pH 3-8.4; 1.2-1,167 µM NH_4_^+^, listed in Figure S1) and temperatures up to 97°C [19,20,26,27,30]. The mere detection of archaeal *amoA*-like genes in an environment does not always prove nitrifying activity of the respective microbes, not even if these genes occur in high copy numbers and are transcribed [6]. However, our ammonia-oxidizing enrichment of a thaumarchaeote from this cluster from a thermal spring and the global distribution of highly similar *amoA*-like genes in hot environments indicate that this particular lineage has at least the metabolic capability to perform nitrification in these habitats.

Furthermore, we established a clone library of the *hcd* gene encoding archaeal 4-hydroxybutyryl-CoA dehydratase, a key enzyme of the 3-hydroxypropionate/4-hydroxybutyrate cycle for autotrophic CO_2_ fixation [50]. RFLP analysis of 25 hcd clones revealed two different band patterns, and six clones representing either pattern were sequenced and phylogenetically analyzed (Figure 2D). The sequences were highly similar (≥99.5% nucleotide identity) to each other, but showed only moderate similarity (<77% nucleotide identity) to thaumarchaeotal *hcd* sequences from other environments and cultures. However, it has to be considered that existing databases for this gene are much less exhaustive than 16S rRNA and *amoA* gene collections. 

Taken together, the analyses of four molecular marker genes demonstrated that culture N4 contained a novel ammonia-oxidizing thaumarchaeote that is only distantly related to other cultured AOA.

### Electron microscopy

**Figure 3:**
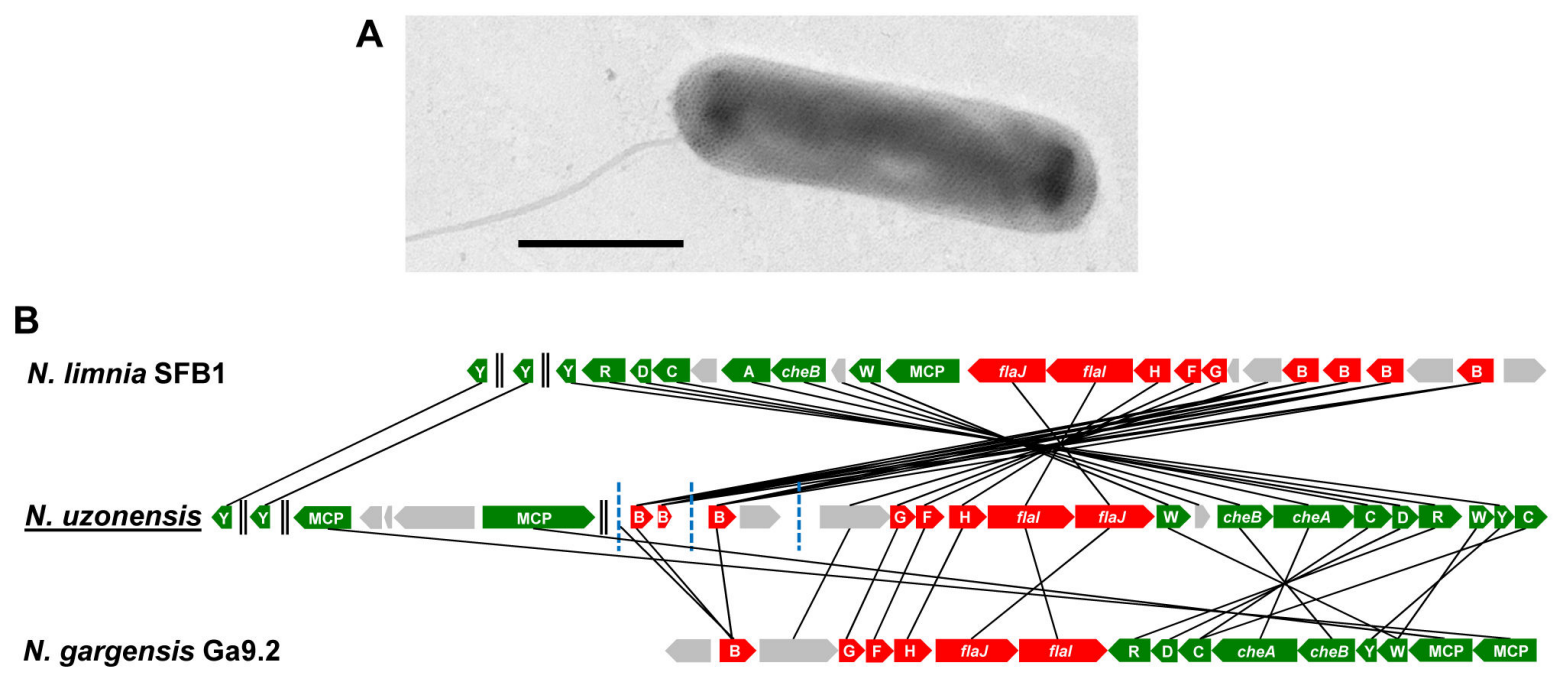
Findings supporting flagellation of “*Ca*. Nitrosotenuis uzonensis” (A) Intact cell stained with uranyl acetate, showing a subpolar tail-like cell projection, possibly a flagellum, and a network of hexagonal structures on the cell surface. (B) Organization of flagellar- and chemotaxis-associated genes in its genome. Genes encoding parts of the archaeal flagellum (*fla* genes) and chemotaxis apparatus (*che* genes) are given in red and green, respectively, whereas other genes are shown in grey. For details on these genes please refer to Table S8 in File S1. Black double bars indicate separate genomic regions, while blue bars display contig ends. MCP, putative methyl-accepting chemotaxis protein.

When electron microscopic analyses were performed, the enrichment still contained cells that were detectable by FISH using the EUB338 probe set. However, the majority of the cells did not hybridize with these probes and most of them were thin rods. Some of these rods were clearly assigned to the novel AOA by CARD-FISH with probes Arch915 and Cren512 (Table S2 in File S1), but the hybridization signal was very weak and many cells neither hybridized with the bacterial nor the archaeal probes. Consistently, by electron microscopy thin rods were observed as the dominant morphotype in the enrichment and we thus assumed that these cells are the novel AOA. These cells appeared as slender, straight rods with a diameter of 0.2-0.3 µm and a length of 0.4-1.7 µm. One or two polar to subpolar tail-like cell projections were observed, which we assume to be flagella (Figure 3A). In whole cell preparations stained with uranyl acetate a network of hexagonal structures, typical for archaeal paracrystalline surface layers [46], was visible (Figure 3A). 

### Genome reconstruction of the novel archaeon

The near-complete genome of the novel, moderately thermophilic AOA was reconstructed using an environmental genomics approach. At the time of sampling for genome sequencing, thaumarchaeotes constituted ~50% of cells in culture N4 according to visual quantification after catalyzed reporter deposition (CARD) FISH using probes Arch915 and Cren512 (Table S2 in File S1), but this number was likely an underestimate of the actual degree of enrichment as the CARD-FISH signals were very weak indicating either a very low ribosome content of the AOA or problems with cell permeabilization.

The genome of the novel AOA is represented by a single scaffold containing 14 contigs with a total length of 1,649,125 bp, mean sequence coverage of 39.4 and an average GC-content of 42.2%. Other key features of the genome are listed in Table 1 and Figure S2. The near completeness of the genome is indicated by the presence of all 138 clusters of orthologous groups (COGs) of proteins that are encoded in all sequenced archaeal genomes (archCOGs; Table S3 in File S1). The 16S rRNA, *amoA, amoB*, and *hcd* gene sequences in the genome were highly similar (>99% nucleotide identity for each gene) to the sequences retrieved from the aforementioned clone libraries. No sequence indicative of the presence of ammonia or nitrite oxidizing bacteria or other archaea was found in the metagenome.

**Table 1:** Overview of key features of the “*Ca*. Nitrosotenuis uzonensis” genome.

Genome size	1,649,125 bp
Average GC-content	42.25 %
Number of scaffolds	1
Number of contigs	14
Number of genomic objects (CDS, fCDS, tRNAs, rRNAs)	1,999
Number of CDS	1,958
Number of CDS with predicted function	411 (21.0%)
CDS density	1.19 CDS/kb
Average CDS length	766.3 bp
Average intergenic length	98.5 bp
Protein coding density	90.2 %
Number of 16S-23S rRNA operons	1
Number of 5S rRNAs	1
Number of tRNAs	38
Number of *amoA*, *amoB*, *amoC* genes	1 / 1 / 2
Number of *accA*/*pccB* and *hcd* genes	1 / 1

CDS, predicted coding sequences; fCDS, fragmented CDS

### Ammonia oxidation

The genome of the enriched thaumarchaeote encodes all proteins that have been hypothesized to be involved in the oxidation of ammonia and subsequent electron transport in *N. maritimus* SCM1 (Table S4 in File S1) [51]. Considering the distant phylogenetic relationship and different habitat of the novel thaumarchaeote compared to the other genome-sequenced AOA, the conservation of this protein set in the novel and previously available genomes [38,43,51-54] supports the proposed key functional role in the metabolism of these organisms. Like the other genome-sequenced AOA, the analyzed thaumarchaeote possesses single copies of the *amoA* and *amoB* genes, whereas two copies of *amoC* (96.3% amino acid identity) are present. The *amo* genes are clustered with an arrangement identical to the order in the genomes of *N. maritimus* SCM1, “*Ca*. N. limnia” SFB1 and “*Ca*. N. koreensis” MY1 (*amoA*, *amoX*, *amoC*, *amoB*) [38,51,52,55].

### Transport and use of alternative substrates

While two AmtB-type ammonia transporters were identified (Tables S4 and S5 in File S1), we found no genes coding for a urea transporter or a urease, which would enable the organism to conserve energy by oxidizing ammonia derived from the hydrolysis of urea. This finding is consistent with the absence of such genes in *N. maritimus* SCM1, “*Ca*. Nitrosopumilus Salaria” and “*Ca*. N. limnia” sp. [38,43,51-53]. In contrast, “*Ca*. Cenarchaeum symbiosum” A was reported to harbour all essential genes for ureolysis [56], but experimental support for this as well as its ammonia-oxidizing activity is lacking so far. However, ureolytic activity has been demonstrated in AOA from the *Nitrososphaera* cluster [44,54]. In addition, in the AOA enriched in our study, no genes for the uptake or utilization of cyanate as potential energy source, as recently hypothesized for “*Ca*. N. gargensis” [54], were found in the genome.

In contrast to the genome of “*Ca*. N. gargensis”, the archaeon enriched in this study only encodes a limited set of heavy metal transporters comparable to those of *N. maritimus* and ”*Ca*. N. limnia” (Table S5 in File S1). Unfortunately, no data on heavy metal concentrations in the thermal spring water used for inoculation are available and it remains unknown whether heavy metal exposure for strain N4 is lower than for “*Ca*. N. gargensis” from the Garga hot spring.

The genome of strain N4 encodes transporters of amino acids and oligo/dipeptides, as well as putative alanyl-, methionyl/ and leucyl-aminopeptidases (Table S5 in File S1). This suggests that strain N4 might be capable of mixotrophic growth on these substrates or at least can utilize them as an additional source of nitrogen. No systems for the uptake or degradation of glycerol or specific sugars, as found in other AOA [51,54], could be identified. Consistent with the finding that the addition of organic acids (formate, acetate, pyruvate, and succinate) to the culture did not result in a change of ammonia oxidation rates, no transporters for these compounds could be identified in the genome.

### Carbon metabolism

Recent data suggest that AOA grow autotrophically by fixing inorganic carbon via a modified 3-hydroxypropionate/4-hydroxybutyrate pathway (3HP/4HB) [50,51,56]. Two key enzymes of this cycle are 4-hydroxybutyryl-CoA dehydratase (Hcd) and acetyl-CoA/propionyl-CoA carboxylase (AccA/PccB), which recently have been used to study the distribution and abundance of autotrophic thaumarchaeotes in the environment [57-59]. The successful amplification of archaeal *hcd* gene sequences from culture N4 (Fig. 2D), which contained bicarbonate/carbon dioxide as the sole source of carbon, already indicated that the novel AOA uses this pathway for the fixation of inorganic carbon. This hypothesis was corroborated by the presence of a near complete 3HP/4HB cycle in the genome of the enriched AOA (Table S6 in File S1). This finding supports the proposed wide distribution of a 3HP/4HB-like cycle in AOA and is in agreement with the observed thermotolerance of this carbon fixation pathway [60]. It should be noted that the primers recently developed for the detection of archaeal *hcd* [57] and *accA* [61] genes in environmental samples contain up to ten nucleotide mismatches to the respective genes of strain N4 and of “*Ca*. Nitrososphaera gargensis”. Although *hcd* gene fragments of strain N4 were amplifiable by using the published *hcd*-targeted primers and recommended PCR conditions, both primer sets could introduce strong biases in molecular analyses of thaumarchaeotal community structure. 

Strain N4 encodes a cofactor F_420_-dependent NADP oxidoreductase as well as all key genes for F_420_-biosynthesis (Table S7 in File S1) that have recently been identified in all other available AOA genomes [54]. However, in contrast to “*Ca*. N. gargensis” [54], but in accordance to all other AOA, the genome of strain N4 does not encode a F_420_-dependent glucose-6-phosphate dehydrogenase. While the role of F_420_ in AOA is yet unknown, this cofactor serves as electron carrier in methanogens [62] and in bacteria it has been shown to be involved in the protection from oxidants [63] and reactive nitrogen species [64]. In contrast to “*Ca*. N. gargensis”, we could not microscopically observe the characteristic blue fluorescence of this cofactor in a highly active culture of strain N4. However, in this context it is important to note that F_420_ content also varies considerably in different methanogens [65] .

As all other genome-sequenced AOA, strain N4 encodes a polyhydroxyalkanoate synthase, the key enzyme for the production of polyhydroxyalkanoate (PHA), which most probably starts from the 3HP/4HB-cycle intermediate 3-hydroxybutyryl-CoA [54]. Similar to all other thaumarchaeotes, with the exception of “*Ca*. N. gargensis” [54], the enzyme is encoded by a single locus of two partly overlapping genes (*phaCE*, encoded by Nituzv1_30469 and Nituzv1_30470). As in other AOA, canonical PHA depolymerases are absent from the strain N4 genome. In contrast to “*Ca*. N. gargensis”, Raman micro-spectroscopic analyses of strain N4 cells did not reveal signature bands of polyhydroxyalkanoates (data not shown), but this does not exclude the production of this storage polymer under different growth conditions.

### Flagellation and chemotaxis

The novel AOA encodes a number of genes that have been associated with archaeal chemotaxis and flagellar assembly (Table S8 in File S1; Figure 3B). It comprises the full gene set required for the synthesis of an archaeal flagellum, and our observation of flagella-like structures during EM analyses (Figure 3A) suggest that these genes are actively expressed in the enrichment culture. Homologous genes are mostly absent from the genomes of *N. maritimus* SCM1 and “*Ca*. C. symbiosum” A, but were identified in “*Ca*. N. limnia” sp. as well as in the moderate thermophile “*Ca*. N. gargensis”, and are typically clustered in a single region (Figure 3B) [38,53,54]. As observed for the genomes of “*Ca*. N. limnia” species [38,53], the novel genome harbours more than one copy of *flaB*. The exact number is unknown, because the respective gene fragments are located at contig ends (Figure 3B). It should, however, be noted that in “*Ca*. N. limnia” sp. the four *flaB* genes are clustered in one locus, suggesting that the gaps between the respective contigs in the strain N4 genome are likely to be only very small.

In the genome of strain N4, the flagella genes are co-localized with a gene cluster harbouring the essential gene set for chemotaxis (i.e. *cheA*, *cheB*, *cheC*, *cheD*, *cheR*, *cheW*, *cheY*; Figure 3B, Table S8 in File S1). Furthermore, the genome contains 13 putative histidine kinases as well as 8 response regulator-like proteins (Table S8 in File S1). Chemotaxis and motility likely are beneficial to AOA living in heterogeneous habitats where they are exposed to shifts in substrate concentrations and/or to detrimental conditions. In contrast, these features seem to be less important in habitats of AOA lacking such genes like the open ocean, where *N. maritimus*-like archaea reside [15,66,67], or sponge tissue in which “*Ca*. C. symbiosum” thrives [68]. As most other genome-sequenced thaumarchaeotes (the only exception is "Ca. N. gargensis" Ga9.2) [54], strain N4 does not encode gas vesicles.

### Potential cell division system

As other genome-sequenced thaumarchaeotes, the enriched AOA encodes homologues of both crenarchaeotal (*cdvABC*) and euryarchaeotal/bacterial (*ftsZ*) cell division machinery components (Table S9 in File S1) [5,69]. In *N. maritimus* the ESCRTIII-related Cdv-system has been demonstrated to be the primary division system, while the role of FtsZ remains unknown [70,71]. Recently, an additional distant tubulin homologue was identified in the genomes of each “*Ca*. N. limnia” SFB1 and “*Ca*. Nitrosoarchaeum koreensis” [52] and named “artubulin” [72]. It was suggested that these hypothetical thaumarchaeotal proteins may be evolutionary ancestral to eukaryotic tubulin [72], but the functionality of this protein as well as its sub-cellular localization is yet untested. Interestingly, the genome of strain N4 also encodes an “artubulin”-like protein (Table S9 in File S1) with ~79% amino acid identity to the homologues of the *Nitrosoarchaeum* species. The predicted protein contains all amino acid residues that are conserved in the majority of tubulins including the proposed “artubulins”, but which are not shared by the majority of FtsZ sequences [72]. However, in contrast to the *Nitrosoarchaeum* species, strain N4’s “artubulin”-like protein is not found in proximity to one of its Snf7/CdvB-encoding genes (Table S9 in File S1) [52,53,72]. 

### Genomic plasticity of AOA

The obtained genome sequence was compared to the genomes of its closest relatives, *N. maritimus* SCM1*,* “*Ca*. N. limnia” SFB1 and “*Ca*. C. symbiosum” A, to search for regions of genomic plasticity (RGP) that mainly contain genes specific for the novel thaumarchaeote. This analysis revealed numerous RGP with a total length of ~503 kb (Figure S2). Most genes in these regions encode hypothetical proteins or proteins of unknown function and lack homologs in the other AOA genomes, suggesting that they might be involved in habitat-specific adaptations such as life at elevated temperatures. The comparative genomic analysis between strain N4 and *N. maritimus* SCM1 also showed that 1,196 coding sequences (CDS; 61.1%) of the novel thaumarchaeote and 1,205 CDS (67.1%) of *N. maritimus* SCM1 are organized in 106 syntenic regions (syntons) with an average length of 11 CDS. A genome-wide dot plot (Figure S3A) confirmed a high degree of genomic synteny between these two AOA, which was also found in comparison to “*Ca*. N. limnia” SFB1 (Figure S3B). When compared to “*Ca*. C. symbiosum” A, in total 157 syntons were detected that contained 1,113 CDS (56.8%) of the novel thaumarchaeote and 1,125 CDS (55.5%) of “*Ca*. C. symbiosum” A. However, these syntons were shorter with an average length of 7 CDS and much more scattered, with genome-scale synteny being very low (Figure S3C). A low overall synteny to “*Ca*. C. symbiosum” A has also been reported for genomes of other AOA of the *Nitrosopumilus* cluster [38,51], indicating that numerous genomic rearrangements must have occurred during the evolution of “*Ca*. C. symbiosum” whose lifestyle as sponge symbiont differs markedly from that of free-living AOA.

### A novel thaumarchaeotal species

The phylogenetic analyses of four marker genes (Figure 2) indicate that the enriched archaeon represents a new species, which can clearly be distinguished from all previously described AOA. This gains strong support from average nucleotide identity (ANI) analyses, which were based on the genome sequences of this organism and of its closest sequenced relatives, *N. maritimus* SCM1 and "*Ca*. C. symbiosum" A. The obtained ANI values of 67.1% (compared to *N. maritimus* SCM1) and 64.8% (compared to "*Ca*. C. symbiosum" A) are far below the threshold of 95-96%, which corresponds to DNA-DNA hybridization values of 60-70% and has been suggested as the ANI boundary for circumscribing archaeal and bacterial species [73]. Tetranucleotide signature frequency correlation coefficients, also inferred from these three genome sequences, were 0.58 (compared to *N. maritimus* SCM1) and 0.55 (compared to "*Ca*. C. symbiosum" A). In general, much higher correlation coefficients (>0.99) correspond to ANI values above 96% [73]. Thus, multiple lines of evidence support that strain N4 is a new thaumarchaeotal species.

### Provisional classification and conclusion

In this study we demonstrate that strain N4, which we obtained in our moderately thermophilic enrichment culture, is a thaumarchaeote of the *Nitrosopumilus* cluster that is responsible for the observed oxidation of ammonia. We propose the following *Candidatus* status for this archaeon: “Nitrosotenuis uzonensis” fam et. gen. et sp. nov. 

#### Etymology

Nitrosus (Latin masculine adjective), nitrous; tenuis (Latin masculine adjective), small/slender; uzonensis (Latin neutrum genitive), from Uzon.

#### Locality

A terrestrial thermal spring located in the Uzon caldera on the Kamchatka peninsula, Russia.

#### Diagnosis

A chemolithoauotrophic ammonia oxidizer affiliated with the *Nitrosopumilus* cluster as defined by [48] of the phylum *Thaumarchaeota* within the domain *Archaea*.

In addition to “*Ca*. N. yellowstonii” [18] of the *Nitrosocaldus* cluster and “*Ca*. N. gargensis” [22] of the *Nitrososphaera* cluster, this archaeon is the third identified species of AOA growing at elevated temperatures. “*Ca*. Nitrosotenius uzonensis” is the first cultured representative of *Nitrosopumilus*-cluster AOA with worldwide distribution in terrestrial geothermal habitats. Furthermore, as evidenced by the phylogenetic clustering of our AOA with the recently cultivated strains “AOA-AC5” and “AOA-DW” [39], this phylogenetic clade is not confined to habitats with elevated temperature, but also occurs in freshwater sediments. Their impact on biogeochemical cycling in these systems, however, has yet to be determined.

## Materials and Methods

### Enrichment culture

An ammonia-oxidizing enrichment culture, inoculated with water and sediment from a terrestrial thermal spring located in the Uzon caldera on the Kamchatka peninsula (Russia), was established in 2006 and maintained for seven years. A temperature of 45°C and a pH of 6.5 were measured at the sampling site. The concentration of ammonium was 34.4 µM, whereas nitrite and nitrate were not detectable at the time of sampling [45]. At an early stage of the enrichment, different incubation temperatures were tested (28-52°C) in parallel to determine the optimal conditions for ammonia oxidation. The culture was grown aerobically in static 300 ml Erlenmeyer flasks or static 250 ml (Schott AG, Mainz, Germany) flasks. The cells were grown in medium containing (per litre) 54.4 mg KH_2_PO_4_, 74.4 mg KCl, 49.3 mg MgSO_4_ ×7H_2_O, 584 mg NaCl, 33.8 µg MnSO_4_, 49.4 µg H_3_BO_3_×7H_2_O, 43.1 µg ZnSO_4_×7H_2_O, 37.1 µg (NH_4_)_6_Mo_7_O_24_×4H_2_O, 97.3 µg FeSO_4_×5H_2_O, and 25.0 µg CuSO_4_×2H_2_O. Furthermore, either 147 mg CaCl_2_ or 4.0 g CaCO_3_ L^-1^ (which partially precipitated) was added. At late stages of the enrichment, only CaCO_3_-based medium was used. Typically, fresh medium contained 1 mM NH_4_Cl. In the case of CaCl_2_-medium, the pH was adjusted to 7.4-7.8 by adding 1% (w/v) NaHCO_3_ solution. To further enrich for AOA, consecutive application of vancomycin (100 mg L^-1^) and streptomycin (50 mg L^-1^) was performed, which was followed by inoculation into fresh medium (10 vol%). Sodium salts of either formate, acetate, pyruvate, or succinate were added (20 mg L^-1^) to test whether strain N4 used these organic substrates. The enrichment culture was regularly transferred by inoculation of flasks containing fresh medium with 10 vol% of the culture. The concentrations of nitrite and nitrate were regularly measured by using Merckoquant test stripes (Merck, Vienna, Austria). For recording substrate turnover, ammonium, nitrite, and nitrate concentrations were precisely measured as described recently [22,74].

### Amplification, cloning, sequencing, and phylogenetic analyses of 16S rRNA, amo, and hcd genes

DNA was extracted from enrichment culture N4 either by phenol/chloroform-extraction including a bead-beating step (30 s, 6 m/s; adapted from [75]) or by using the PowerSoil kit (MOBio Laboratories, Darmstadt, Germany) according to the instructions of the manufacturer. PCR mixtures contained 2 mM MgCl_2_ and 1 U of Taq-polymerase (Fermentas Life Sciences, St. Leon-Rot, Germany) in combination with 50 pmol of the respective oligonucleotide primers. In all PCR 30 cycles were used to amplify the target DNA and a final elongation step of 10 min at 72 °C was performed. A list of all primers with the applied annealing temperatures is provided in Table S1 in File S1. Amplification of bacterial *amoA* genes was attempted by using primers amoA1F and amoA2R [76], and a plasmid carrying an *amoA* gene of *Nitrosomonas* sp. as template was used as positive control. Cloning and sequencing were performed as described elsewhere [22]. The cloned 16S rRNA and *hcd* genes were screened, prior to sequencing, by RFLP analysis using the restriction endonucleases MspI and AluI (16S rRNA) or RsaI (*hcd*) (Fermentas Life Sciences, St. Leon-Rot, Germany), whereas *amoA* and *amoB* clones were randomly chosen for sequencing without RFLP analysis. For each unique RFLP pattern at least one representative clone was sequenced. Phylogenetic analyses were conducted using the programs ARB [77] and Phylip [78] with comprehensive databases. Automatic tools included in ARB were used to align sequences to the respective in-house databases. All alignments were manually refined. A 16S rRNA consensus tree was reconstructed based on Maximum-Likelihood (ML) calculation (using the Hasegawa, Kishino and Yano substitution model) and by collapsing all nodes with parsimony bootstrap (100 iterations) support below 70%. A sequence filter considering only positions conserved in ≥50% of all thaumarchaeotal and crenarchaeotal sequences in our database was used (resulting in 1,360 alignment positions for the tree calculations) and only sequences ≥1,300 nucleotides in length were considered. Shorter sequences were added later via the “parsimony interactive” tool within ARB without changing the overall topology of the tree. For the analyses of AmoA (176 considered amino acids), AmoB (152 amino acids) and Hcd (125 amino acids) sequences, Fitch-Margoliash distance trees (7 jumbles, Kimura substitution model, randomized sequence order) were reconstructed using Phylip [78] without the use of filters. In addition, parsimony bootstrapping (1,000 iterations) analyses were conducted using ARB, and its values projected onto the Fitch tree.

### FISH and CARD-FISH

For the *in situ* detection and visualization of bacterial and archaeal cells in enrichment culture N4, FISH and CARD-FISH were performed as described elsewhere [22] with a hybridization time of 3 h and the probes listed in Table S2 in File S1. Fluorescein- and Cy3-labelled tyramides were synthesized according to Pernthaler et al. [79] and diluted 1:1,000 in amplification buffer. After FISH or CARD-FISH, cells were stained with 4’,6’-diamidino-2-phenylindole (DAPI, 1 µg ml^-1^) and were microscopically analyzed using a Zeiss LSM 510 confocal laser scanning microscope and the included software. The absence of unspecific fluorescence after CARD-FISH was confirmed by using probe NON-EUB (Table S2 in File S1) that does not bind to any known organism.

### Electron microscopy

Negative staining of whole cells was performed using 1% uranyl acetate in distilled water for 10-20 s. Micrographs were recorded using a JEM-100CX II transmission electron microscope (JEOL, Tokyo, Japan).

### Metagenomic sequencing and analyses

For obtaining biomass for metagenome sequencing, culture N4 was grown aerobically in 300 ml Erlenmeyer flaks as described above. Biomass was harvested by centrifugation (11,000 rpm, 10 min, room temperature) and frozen (-20 °C) until phenol/chloroform-based DNA extraction (see above) was performed. The genomic DNA was submitted to mechanical fragmentation through nebulization (hydroshear) with a 3 kb mean fragment size. After recircularization using an adapter, the DNA was fragmented using an E210 ultrasound DNA-shearer (Covaris, Woburn, MA), and 600 bp fragments containing the adapter were selected for sequencing. Three quarters of a 454 Titanium mate-paired run were performed, generating ~250 Mb of sequence in 796,942 reads. In addition, Illumina (GA-IIx) single read sequencing was performed, generating 34,689,904 reads of 36 bp each. The 454-reads were subjected to assembly by using the Newbler software package, generating 739 contigs on 41 scaffolds with a cumulative size of ~5.5 Mb. The largest of these scaffolds, containing 14 contigs with a total length of 1,649,125 bp and a mean contig coverage of 39.4, represents the genome of strain N4. The Illumina reads were used to correct 454 sequencing errors as described by Aury et al. [80]. Corrected contigs assembled using the scaffold description were used to generate the final sequence. The MaGe annotation platform (Vallenet et al., 2006) was used for CDS prediction, automatic annotation, and manual annotation refinement according to guidelines as recently described [81]. Statistics about syntons in compared genomes were generated by the respective tools of MaGe, where the gap parameter, which represents the maximum number of consecutive genes that are not involved in a synton, was set to five genes. Regions of genomic plasticity were identified by the respective tool of MaGe based on synteny breaks in the compared genomes and features of potential lateral gene transfer events, such as tRNA hotspots and DNA compositional biases, in the genome of strain N4. Genome-scale dot plots (Figure S3) were generated by using the PROmer and mummerplot programs contained in the MUMmer software package (version 3.22) [82] in combination with Gnuplot (version 4.4.3). The circular genome map (Figure S2) was generated using the software Circos (version 0.54) [83]. Average nucleotide identity (ANI) values were calculated, based on whole-genome sequences of AOA and on the BLAST algorithm, by using the software JSpecies [73] with the default parameters. The same software was applied to determine tetranucleotide signature frequency correlation coefficients for the AOA genomes.

### Sequence deposition

The genome sequence of “*Ca*. Nitrosotenuis uzonensis” has been deposited at the European Nucleotide Archive with the master reference number CBTY000000000.

## Supporting Information

Figure S1 **Phylogenetic analysis of a selection of AmoA sequences obtained from geothermal sites that are closely related to the sequence of “*Ca*. Nitrosotenuis uzonensis”.** The tree shows sequences affiliated with the *Nitrosopumilus* cluster, while sequences belonging to the *Nitrososphaera, Nitrosotalea* and *Nitrosocaldus* clusters have been used as outgroup. If available, temperature, pH and sample type are indicated for clones obtained from geothermal systems (in red font). Circles on tree nodes indicate parsimony bootstrap support ≥90%. Numbers in parentheses indicate the number of sequences within a group. The scale bar equals 10% estimated sequence divergence.
(TIF)

Figure S2 **Circular representation of the “*Ca*. N. uzonensis” chromosome.** Contigs are separated by white space in all rings. Predicted coding sequences (rings 1+2), RNA genes (ring 3), regions of genomic plasticity compared to *N. maritimus* SCM1 (ring 4) or “*Ca*. Cenarchaeum symbiosum” (ring 5), and local nucleotide composition measures (rings 6+7) are shown. Very short features were enlarged to enhance visibility.
(TIF)

Figure S3 **Dot plots representing genome-wide alignments of “*Ca*. Nitrosotenuis uzonensis” to the genomes of** (A) *Nitrosopumilus maritimus* SCM1, (B) “*Ca*. Nitrosoarchaeum limnia” SFB1, and (C) “*Ca*. Cenarchaeum symbiosum” A. Forward matches are shown in red, while reverse matches are shown in blue.
(TIF)

File S1 **Combined file containing tables S1-S10.**
(XLSX)
