# Supplementary figures and images for: Enrichment and Genome Sequence of the Group I.1a Ammonia-Oxidizing Archaeon “Ca. Nitrosotenuis uzonensis” Representing a Clade Globally Distributed in Thermal Habitats
Source: PLoS One. 2013 Nov 20;8(11):e80835. doi: 10.1371/journal.pone.0080835 (PMC3835317; doi:10.1371/journal.pone.0080835)

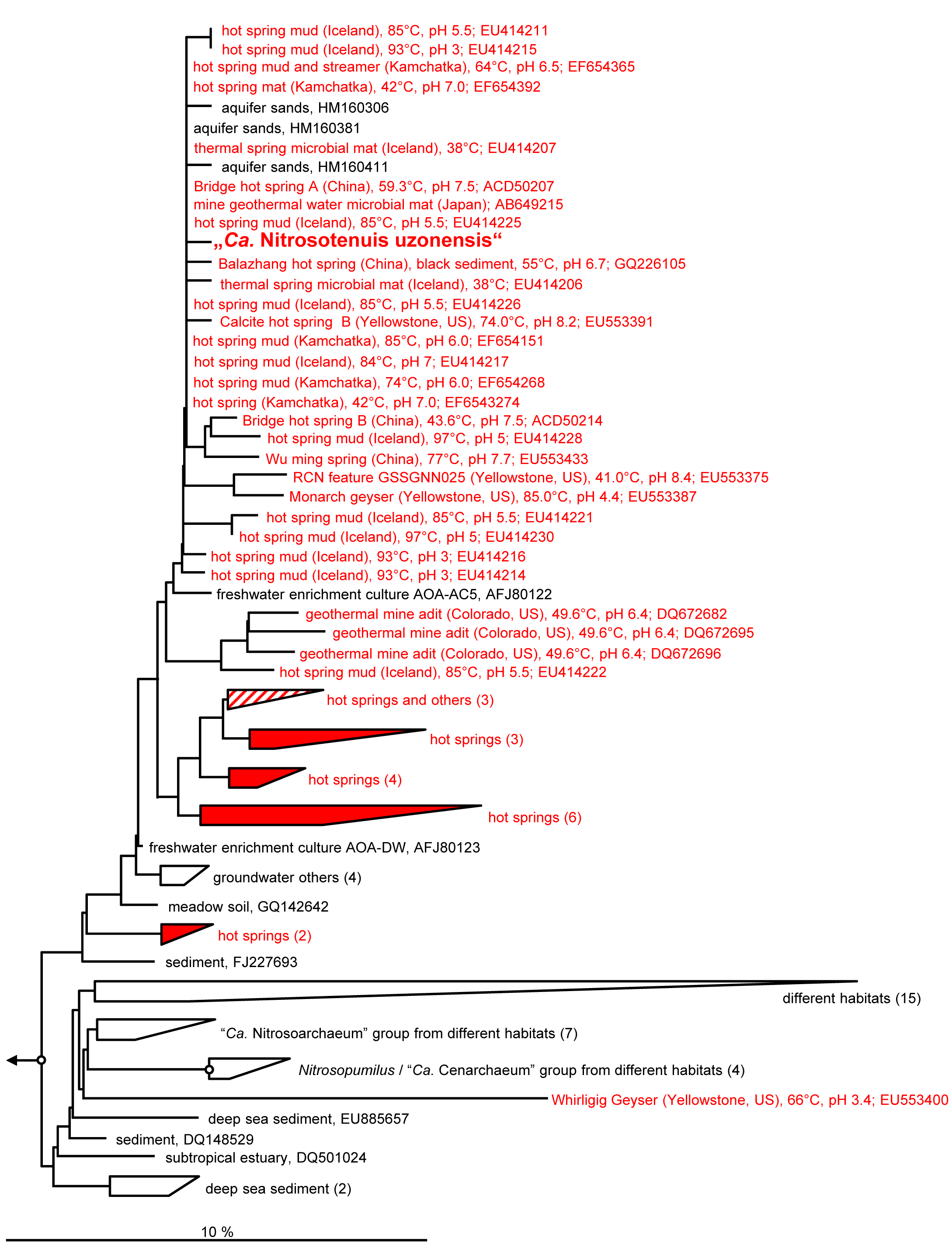

Supplement: Figure S1 — Phylogenetic analysis of a selection of AmoA sequences obtained from geothermal sites that are closely related to the sequence of “Ca. Nitrosotenuis uzonensis”. The tree shows sequences affiliated with the Nitrosopumilus cluster, while sequences belonging to the Nitrososphaera, Nitrosotalea and Nitrosocaldus clusters have been used as outgroup. If available, temperature, pH and sample type are indicated for clones obtained from geothermal systems (in red font). Circles on tree nodes indicate parsimony bootstrap support ≥90%. Numbers in parentheses indicate the number of sequences within a group. The scale bar equals 10% estimated sequence divergence. (TIF) [file pone.0080835.s001.tif]

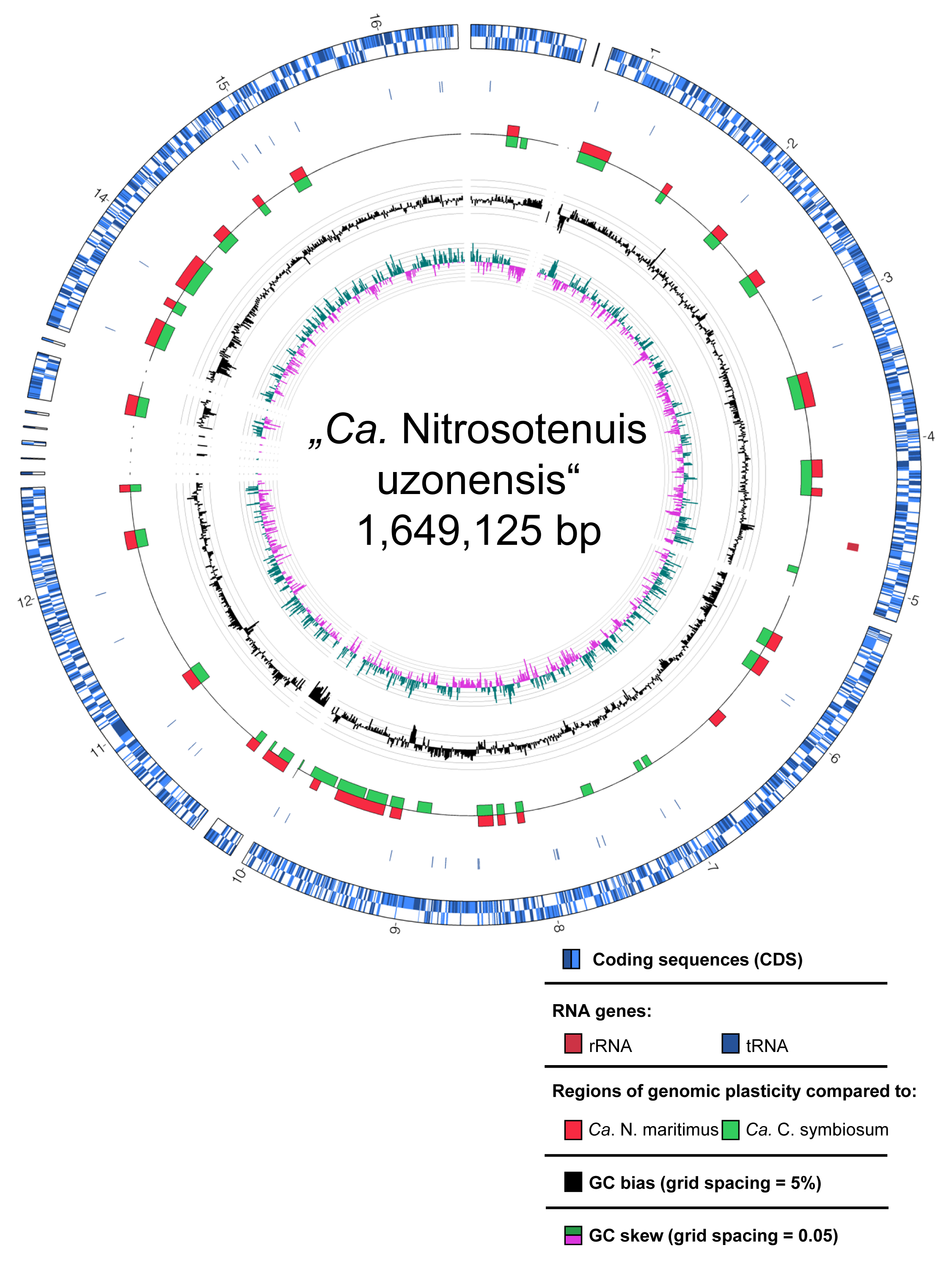

Supplement: Figure S2 — Circular representation of the “Ca. N. uzonensis” chromosome. Contigs are separated by white space in all rings. Predicted coding sequences (rings 1+2), RNA genes (ring 3), regions of genomic plasticity compared to N. maritimus SCM1 (ring 4) or “Ca. Cenarchaeum symbiosum” (ring 5), and local nucleotide composition measures (rings 6+7) are shown. Very short features were enlarged to enhance visibility. (TIF) [file pone.0080835.s002.tif]

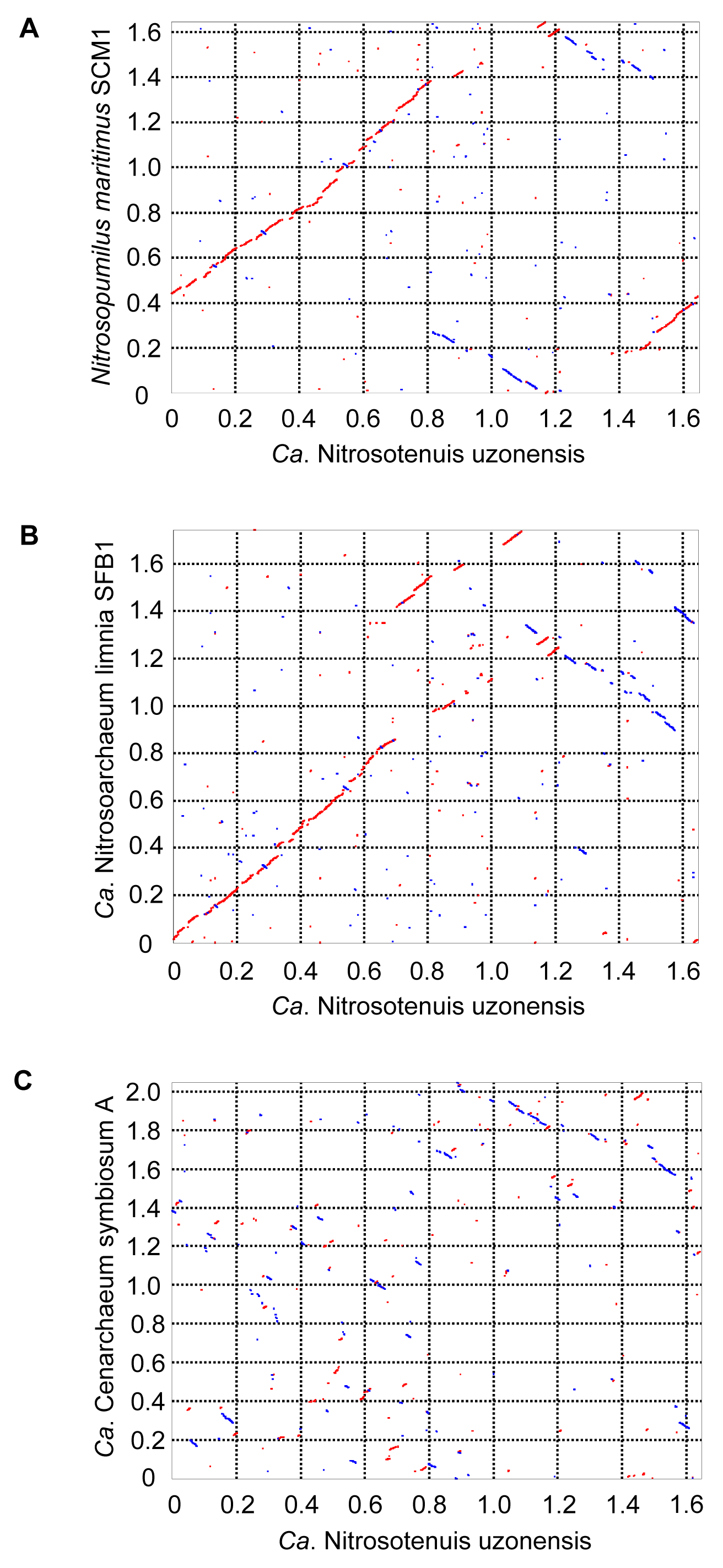

Supplement: Figure S3 — Dot plots representing genome-wide alignments of “Ca. Nitrosotenuis uzonensis” to the genomes of (A) Nitrosopumilus maritimus SCM1, (B) “Ca. Nitrosoarchaeum limnia” SFB1, and (C) “Ca. Cenarchaeum symbiosum” A. Forward matches are shown in red, while reverse matches are shown in blue. (TIF) [file pone.0080835.s003.tif]
